# Hemophagocytic Macrophages in the Peripheral Blood of a Critically Ill Patient With COVID‐19 and RSV Infection

**DOI:** 10.1002/ajh.70243

**Published:** 2026-02-18

**Authors:** Ashik Zala, Ahmed Sadek, Ahmed Elsaid, Ketan C. Patel, Maximiliano Ramia De Cap, Barbara J. Bain

**Affiliations:** ^1^ Department of Haematology Imperial College Healthcare NHS Trust, Hammersmith Hospital London UK; ^2^ Faculty of Medicine, Centre for Haematology St Mary's Hospital Campus of Imperial College London London UK

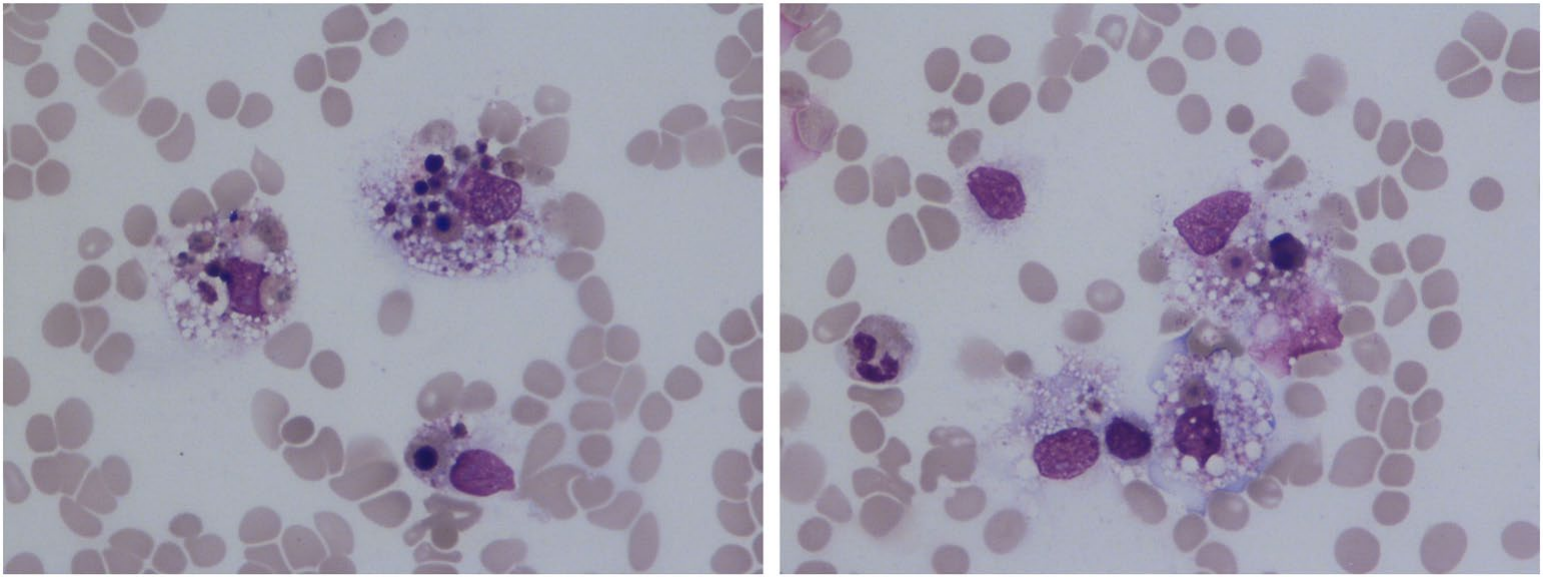

The blood film of critically ill, 47‐year‐old man was examined because of a markedly abnormal blood count. The patient had been intubated and was being ventilated for type 1 respiratory failure secondary to concomitant COVID‐19 and respiratory syncytial virus (RSV) infection on a background of alcohol‐related liver disease with portal hypertension and ascites. He was also requiring continuous renal replacement therapy and vasopressor and corticosteroid support because of septic shock and multiorgan failure. There was marked hyperbilirubinemia (bilirubin 680 μmol/L), hypofibrinogenemia (0.69 g/L), and hyperferritinemia (ferritin peaking at 1617 μg/L). At the time of blood film examination his blood count showed severe pancytopenia, most marked for anemia and thrombocytopenia: hemoglobin concentration 54 g/L, mean cell volume 90.4 fL, white cell count 4.6 × 10^9^/L, and platelet count 9 × 10^9^/L. The neutrophil count was 3.3 × 10^9^/L with marked lymphopenia (0.3 × 10^9^/L). The blood film confirmed lymphopenia and thrombocytopenia and showed normocytic, normochromic red cells, nucleated red blood cells (NRBC), scattered microspherocytes and other schistocytes, and, surprisingly, hemophagocytic macrophages (both images, ×100 objective). The ingested cells included pyknotic cells, recognizable NRBC, and platelets. A bone marrow aspirate and trephine biopsy were subsequently performed and showed hemophagocytosis. A diagnosis of hemophagocytic lymphohistiocytosis was made [1]. The blood count and film abnormalities improved with ongoing supportive care but the patient subsequently died of chronic liver failure aggravated by sepsis.

Hemophagocytosis is classically identified in bone marrow, spleen, or lymph node samples. Its demonstration in the peripheral blood is rare and reflects severe systemic macrophage activation [2]. A hemophagocytic syndrome has been reported previously in COVID‐19 and is likely to have been the trigger in this patient [3].

## Conflicts of Interest

The authors declare no conflicts of interest.

## Data Availability

The data that support the findings of this study are available from the corresponding author upon reasonable request.
